# Supplementary material for: Causes and consequences of sex-chromosome turnovers in Diptera
Source: Evol Lett. 2026 Mar 12;10(3):253–68. doi: 10.1093/evlett/qrag003 (PMC13231274; doi:10.1093/evlett/qrag003)
Supplement: qrag003_Supplemental_Files [file qrag003_supplemental_files.zip › Supplementary_Tables 4.zip!/Supplementary_Tables 4/Table_S2_Enrichment_ME__p_values.pdf]

Table S2. Enrichment of Muller elements. Reported p-values were obtained with Fisher's tests.

Species	Elements > 1.5x	Enrichment_1	P-value_1	Enrichment_2	P-value_2	Additional enrichment (observed/expected, p-value with Fisher's test)
Acroceridae_Acrocera_orbiculus	B (one gene)	5.4340223	0.184			
Anthomyiidae_Pegoplata_infirma	F	104.5989	2.20E-16			
Asilidae_Dysmachus_trigonus	F	36.19896	2.20E-16			
Asilidae_Eutolmus_rufibarbis	F	38.61195	2.20E-16			
Asilidae_Machimus_atricapillus	E,F	2.563992	2.20E-16	7.172812	2.20E-16	
Asilidae_Machimus_rusticus	F	35.31256	2.20E-16			
Asilidae_Tolmerus_cingulatus	F	34.79158	2.20E-16			
Bibionidae_Bibio_marce	F	26.81933	2.20E-16			
Bibionidae_Dilophus_febrilis	F	30.0131	2.20E-16			B (1.095455, p=0.6276)
Bombyliidae_Anthrax_anthrax	C,F	3.565179	2.20E-16	7.560891	2.20E-16	
Bombyliidae_Bombylius_discolor	C,F	3.618171	2.20E-16	8.301612	2.20E-16	
Bombyliidae_Bombylius_major	C,F	3.542789	2.20E-16	8.166327	2.20E-16	
Bombyliidae_Villa_cingulata	C,F	3.454852	2.20E-16	7.863875	2.20E-16	
Calliphoridae_Bellardia_pandia	F	117.7966	2.20E-16			
Calliphoridae_Calliphora_vomitoria	F	114.5889	2.20E-16			
Calliphoridae_Protocalliphora_azurea	F	109.2649	2.20E-16			
Calliphoridae_Stomorhina_lunata	F	110.5635	2.20E-16			
Clusiidae_Clusia_tigrina	D	4.713155	2.20E-16			
Coelopidae_Coelopa_pilipes	F	69.41538	2.20E-16			
Conopidae_Conops_quadrifasciatus	F	71.0905	2.20E-16			
Conopidae_Myopa_tessellatipennis	B	5.070062	2.20E-16			
Conopidae_Myopa_testacea	D,F	4.556923	2.20E-16	4.755176	2.20E-16	
Conopidae_Physocephala_rufipes	No X reported					
Conopidae_Sicus_ferrugineus	A (F assigned to Y)	5.192381	2.20E-16			
Conopidae_Thecophora_atra	B	5.130763	2.20E-16			
Culicidae_Anopheles_albimanus	A,F	3.284167	2.20E-16	3.408605	0.0001102	E (1.146621, p=0.04626)
Culicidae_Anopheles_aquasalis	A,F	3.61946	2.20E-16	6.333112	2.12E-07	
Culicidae_Anopheles_arabiensis	A,F	3.285137	2.20E-16	3.952705	1.70E-07	E (1.130511, p=0.05448)
Culicidae_Anopheles_atroparvus	A,F	3.337299	2.20E-16	3.268431	4.55E-05	E (1.125556, p=0.06907)
Culicidae_Anopheles_bellator	A,F	3.279072	2.20E-16	4.658879	7.71E-07	E (1.202495, p=0.01493)
Culicidae_Anopheles_coluzzii	A,F	3.374499	2.20E-16	2.01311	0.08035	E (1.044514, p=0.5503)
Culicidae_Anopheles_coustani	A,F	3.263742	2.20E-16	4.351655	4.92E-09	E (1.157004, p=0.02108)
Culicidae_Anopheles_cruzii	A,F	3.411808	2.20E-16	5.387065	1.18E-10	E (1.119841, p=0.124)
Culicidae_Anopheles_darlingi	A,F	3.259545	2.20E-16	3.797107	3.97E-06	E (1.010299, p=0.9135)
Culicidae_Anopheles_funestus	A,F	3.42488	2.20E-16	3.379691	1.63E-05	E (1.143846, p=0.02974)
Culicidae_Anopheles_gambiae	A,F	3.240506	2.20E-16	3.176494	0.000118	E (1.095871, p=0.157)
Culicidae_Anopheles_longipalpis	A,F	3.062036	2.20E-16	3.473997	1.34E-06	E (1.113085, p=0.07659)
Culicidae_Anopheles_maculipalpis	A,F	3.304915	2.20E-16	3.90201	1.98E-07	E (1.167851, p=0.01207)
Culicidae_Anopheles_marshallii	A,F	3.302458	2.20E-16	3.745298	2.00E-07	E (1.119875, p=0.07264)
Culicidae_Anopheles_merus	A,F	3.246729	2.20E-16	3.087344	9.42E-05	E (1.182063, p=0.006526)
Culicidae_Anopheles_moucheti	A,F	3.378558	2.20E-16	3.893082	9.47E-08	E (1.158532, p=0.01775)
Culicidae_Anopheles_nili	A,F	3.254199	2.20E-16	3.989018	6.86E-07	E (1.192949, p=0.00476)
Culicidae_Anopheles_parensis	A,F	3.094663	2.20E-16	3.387299	3.59E-06	E (1.105707, p=0.08968)
Culicidae_Anopheles_rivulorum	A	3.037444	2.20E-16			E (1.137879, p=0.06886); F (1.082249, p=0.7852)
Culicidae_Anopheles_stephensi	A,F	3.271483	2.20E-16	4.746479	1.19E-08	E (1.140417, p=0.07197)
Culicidae_Anopheles_vaneedeni	A,F	3.15311	2.20E-16	3.656604	1.23E-06	E (1.100483, p=0.1248)
Culicidae_Anopheles_ziemani	A,F	3.262295	2.20E-16	4.349727	4.96E-09	E (1.156491, p=0.02112)
Diopsidae_Teleopsis_dalmanni	B	4.993742	2.20E-16			
Dolichopodidae_Dolichopus_griseipennis	F	41.46415	2.20E-16			
Dolichopodidae_Poecilobothrus_nobilitatus	F	37.6564	2.20E-16			
Drosophilidae_pseudoobscura_pseudoobscura	A,D	2.818808	2.20E-16	2.759509	2.20E-16	
Drosophilidae_sulfurigaster_albostrigata	A	6.449882	2.20E-16			
Drosophilidae_sulfurigaster_sulfurigaster	A	6.508602	2.20E-16			
Drosophilidae_Drosophila_albomicans	A	6.368059	2.20E-16			
Drosophilidae_Drosophila_ananaese	A (XL), A (XR)	6.378564	2.20E-16	6.429833	2.20E-16	
Drosophilidae_Drosophila_athabasca	A,D	2.82109	2.20E-16	2.744717	2.20E-16	
Drosophilidae_Drosophila_busckii	A	6.440976	2.20E-16			
Drosophilidae_Drosophila_elegans	A	6.436772	2.20E-16			
Drosophilidae_Drosophila_innubila	A	6.467111	2.20E-16			
Drosophilidae_Drosophila_kepulauana	A	6.294079	2.20E-16			
Drosophilidae_Drosophila_Lowei	A,D	2.750047	2.20E-16	2.7094	2.20E-16	
Drosophilidae_Drosophila_mauritiana	A	6.475894	2.20E-16			
Drosophilidae_Drosophila_melanogaster	A	6.372151	2.20E-16			

Drosophilidae_Drosophila_miranda	A (XL), D (XR)	6.287915	2.20E-16	4.339519	2.20E-16	
Drosophilidae_Drosophila_nasuta	A	6.079066	2.20E-16			
Drosophilidae_Drosophila_pallidifrons	A	6.337093	2.20E-16			
Drosophilidae_Drosophila_sechellia	A	6.453619	2.20E-16			
Drosophilidae_Drosophila_subpulchrella	A	6.365122	2.20E-16			
Drosophilidae_Drosophila_teissieri	A	6.491769	2.20E-16			
Drosophilidae_Drosophila_virilis	A	6.396715	2.20E-16			
Dryomyzidae_Dryomyza_anilis	F	75.3782	2.20E-16			
Empididae_Empis_livida	F	42.99091	2.20E-16			
Empididae_Rhamphomyia_laevipes	F	43.03641	2.20E-16			
Heleomyzidae_Suillia_variegata	F	92.30898	2.20E-16			
Hippoboscidae_Ornithomya_chloropus	B.C	2.470209	2.20E-16	2.475026	2.20E-16	
Hippoboscidae_Ornithomya_fringillina	B	5.039729	2.20E-16			
Hybotidae_Hybos_culiciformis	F	49.6451	2.20E-16			
Lauxaniidae_Calliopum_simillimum	F	80.7493	2.20E-16			
Lauxaniidae_Meiosimyza_decipiens	F	71.98148	2.20E-16			
Muscidae_Polietes_domitor	D (one gene)	5.038117	0.1985			
Pallopteridae_Toxonevra_muliebris	F	95.55246	2.20E-16			
Pipunculidae_Nephrocerus_scutellatus	F	49.46298	2.20E-16			
Polleniidae_Pollenia_amentaria	F	104.8361	2.20E-16			
Polleniidae_Pollenia_angustigena	F	115.6673	2.20E-16			
Ptychopteridae_Ptychoptera_albimana	F	29.79738	2.20E-16			
Ptychopteridae_Ptychoptera_contaminata	F	26.48318	2.20E-16			A (1.007631, p=0.9262)
Rhagionidae_Rhagio_lineola	F	30.428	2.20E-16			A (1.026252, p=0.8602)
Rhinophoridae_Melanophora_roralis	F	102.0629	2.20E-16			
Rhinophoridae_Phyto_melanocephala	F	98.97145	2.20E-16			
Sarcophagidae_Sarcophaga_caerulescens	F	111.4776	2.20E-16			
Sarcophagidae_Sarcophaga_roselle	F	103.1513	2.20E-16			
Sarcophagidae_Sarcophaga_subvicina	F	102.4984	2.20E-16			
Sarcophagidae_Sarcophaga_variegata	F	95.21081	2.20E-16			
Sciaridae_Bradysia_coprophila	A,E	1.835514	2.20E-16	2.056566	2.20E-16	
Sciaridae_Pseudolycoriella_hygida	A,E	1.862598	2.20E-16	2.004643	2.20E-16	
Sciomyzidae_Coremacera_marginata	F	72.48094	2.20E-16			
Sciomyzidae_Pherbina_coryleti	F	68.22184	2.20E-16			
Sciomyzidae_Tetanocera_ferruginea	F	73.99778	2.20E-16			
Stratiomydae_Beris_chalybata	F	35.79734	2.20E-16			A (1.005204, p=1)
Stratiomydae_Beris_morrisii	F	36.85472	2.20E-16			
Stratiomydae_Chorisops_tibialis	F	37.71842	2.20E-16			
Stratiomydae_Nemotelus_nigrinus	F	35.98483	2.20E-16			A (1.056674, p=0.6963)
Stratiomydae_Odontomyia_ornata	F	36.72249	2.20E-16			
Syrphidae_Blera_fallax	F	51.24859	2.20E-16			
Syrphidae_Brachypalpus_laphriformis	F	48.89417	2.20E-16			
Syrphidae_Cheilosia_impressa	F	48.49487	2.20E-16			
Syrphidae_Cheilosia_pagana	F	55.42474	2.20E-16			
Syrphidae_Cheilosia_soror	F	48.75456	2.20E-16			
Syrphidae_Cheilosia_urbana	F	50.95009	2.20E-16			
Syrphidae_Cheilosia_vulpina	F	48.1847	2.20E-16			
Syrphidae_Chrysotoxum_bicinctum	F	49.94808	2.20E-16			
Syrphidae_Criorhina_berberina	F	49.04094	2.20E-16			
Syrphidae_Dasysyrphus_albostriatus	F	50.4204	2.20E-16			
Syrphidae_Epistrophe_grossulariae	F	51.47196	2.20E-16			
Syrphidae_Epistrophella_euchroma	F	49.96447368	2.20E-16			
Syrphidae_Episyrphus_balteatus	F	49.78204	2.20E-16			
Syrphidae_Eristalinus_sepulchralis	F	54.75668	2.20E-16			
Syrphidae_Eristalis_arbustorum	F	48.50702	2.20E-16			
Syrphidae_Eristalis_pertinax	F	47.90176	2.20E-16			
Syrphidae_Eristalis_tenax	F	51.73292	2.20E-16			
Syrphidae_Eupeodes_corollae	F	50.37244	2.20E-16			
Syrphidae_Eupeodes_latifasciatus	F	43.51829	2.20E-16			
Syrphidae_Leucozona_laternaria	F	48.5842	2.20E-16			
Syrphidae_Melanostoma_mellinum	F	47.90286	2.20E-16			
Syrphidae_Meliscaeva_auricollis	F	51.18498	2.20E-16			
Syrphidae_Myathropa_florea	F	51.17647	2.20E-16			
Syrphidae_Neoascia_interrupta	F	55.43014	2.20E-16			
Syrphidae_Platycheirus_albimanus	F	46.4371	2.20E-16			
Syrphidae_Rhingia_campestris	F	46.0117	2.20E-16			
Syrphidae_Scaeva_pyrastri	F	48.68382	2.20E-16			
Syrphidae_Sphaerophoria_taeniata	F	50.75315	2.20E-16			

Syrphidae_Syritta_pipiens	F	50.84019	2.20E-16			
Syrphidae_Volucella_inanis	F	46.19034	2.20E-16			
Syrphidae_Volucella_inflata	F	47.68571	2.20E-16			
Syrphidae_Xanthogramma_pedissequum	F	50.76712	2.20E-16			
Syrphidae_Xylota_sylvarum	F	49.43732	2.20E-16			
Tachinidae_Cistogaster_globosa	F	102.8478	2.20E-16			
Tachinidae_Cochliomyia_hominivorax	F	112.4616	2.20E-16			
Tachinidae_Epicampocera_succincta	F	103.5592	2.20E-16			
Tachinidae_Gymnosoma_rotundatum	F	106.2008	2.20E-16			
Tachinidae_Lypha_dubia	F	104.388	2.20E-16			
Tachinidae_Nowickia_ferox	F	117.3275	2.20E-16			
Tachinidae_Tachina_fera	F	100.3038	2.20E-16			
Tachinidae_Tachina_lurida	F	117.2623	2.20E-16			
Tachinidae_Thecocarcelia_acutangulata	F	100.0237425	2.20E-16			
Tachinidae_Thelaira_solivaga	F	86.3495	2.20E-16			
Tephritidae_Anastrepha_ludens	F	93.54354	2.20E-16			
Therevidae_Thereva_nobilitata	F	35.9357	2.20E-16			
Tipulidae_Nephrotoma_appendiculata	F	40.89193	2.20E-16			
Tipulidae_Nephrotoma_flavescens	F	39.40257	2.20E-16			
